# Regional-scale climate-variability synchrony of cholera epidemics in West Africa

**DOI:** 10.1186/1471-2334-7-20

**Published:** 2007-03-19

**Authors:** Guillaume Constantin de Magny, Jean-François Guégan, Michel Petit, Bernard Cazelles

**Affiliations:** 1 Génétique et Evolution des Maladies Infectieuses, UMR CNRS/IRD 2724, 911 avenue Agropolis, BP 64501, 34394 Montpellier cedex 05, France; 2 US ESPACE 140, IRD, 500 rue Jean-François Breton, 34093 Montpellier cedex 05, France; 3 CNRS-UMR 7625, Ecole Normale Supérieure, 46 rue d'Ulm, 75230 Paris cedex 05, France; 4 IRD, UR GEODES, 93143 Bondy, France

## Abstract

**Background:**

The relationship between cholera and climate was explored in Africa, the continent with the most reported cases, by analyzing monthly 20-year cholera time series for five coastal adjoining West African countries: Côte d'Ivoire, Ghana, Togo, Benin and Nigeria.

**Methods:**

We used wavelet analyses and derived methods because these are useful mathematical tools to provide information on the evolution of the periodic component over time and allow quantification of non-stationary associations between time series.

**Results:**

The temporal variability of cholera incidence exhibits an interannual component, and a significant synchrony in cholera epidemics is highlighted at the end of the 1980's. This observed synchrony across countries, even if transient through time, is also coherent with both the local variability of rainfall and the global climate variability quantified by the Indian Oscillation Index.

**Conclusion:**

Results of this study suggest that large and regional scale climate variability influence both the temporal dynamics and the spatial synchrony of cholera epidemics in human populations in the Gulf of Guinea, as has been described for two other tropical regions of the world, western South America and Bangladesh.

## Background

Epidemics of new and old infectious diseases periodically emerge and these emergences reveal the complex dynamical relationships among humans, pathogens and the environment [1,2]. Connections between weather, climate and diseases are well established [3], with many diseases occurring during certain seasons or erupting from unseasonable flood or drought conditions [4]. With new concerns about global warming, accompanied by greater climate variability, many recent studies have focused on disease fluctuations related to interannual climate oscillations (e.g., El Niño) (see [3,5-8]). One of the major underlying questions of these recent studies is: Are climatic oscillations that occur at medium or low time frequency responsible for global patterns of recent reemergence of disease?

Evidence for influence of climate on cholera dynamics in Asia (Bangladesh) [5-7,9,10] and South America (Peru) [6,11] has been published. Cholera, an ancient and devastating acute diarrheal illness caused by the ingestion of toxigenic *Vibrio cholerae*, occurs in widespread epidemics that remain a major public health problem in many developing countries, most often localized in the intertropical belt [12,13]. Studies usually have focused on the influence of climate on cholera dynamics across regions of cholera endemicity, mainly because they can provide environmental or climatic factors that promote epidemics through analysis of long-term historical records [5-7,14,15]. In these regions, cholera dynamics display regular seasonal cycles and pronounced interannual variability. In Bangladesh, as in Peru, nonstationary links have been shown with climate interannual variability (e.g., the El Niño event that occurs every 3–7 years) [5,7,16-18].

The question of what interannual climate variability triggers the disease dynamics patterns in non-endemic cholera regions in Africa, however, remains unanswered. Analyses of long-term climatic and epidemiological data allow exploration of this issue at both the local (e.g., country) and regional scale (e.g., contiguous coastal countries). Africa appears to be the continent most affected by the disease, with more than 95,000 cases reported in 2004 [12].

**Figure 1:**
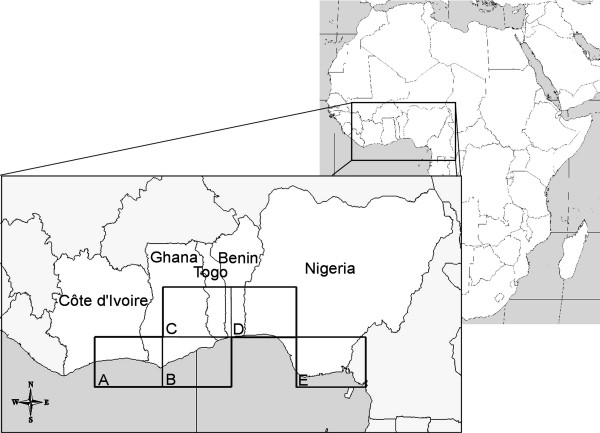
**Map of the five West-African countries included in this study and selected areas of rainfall time series**. Black squares represent the pixel of rainfall, 2.5 decimal degrees of latitude by 3.75 decimal degrees of longitude: (A) Cotiv021, (B) Ghan132, (C) Ben270, (D) Ben201 and (E) Nige037.

In this study, we provide a review of the spatiotemporal patterns of cholera in five African countries on the tropical Atlantic coast of Africa–Côte d'Ivoire, Ghana, Togo, Benin and Nigeria–shown in Figure 1. We address the role of climate interannual variability on both global and local scales (i.e., Indian Oscillation Index (*IOI*) and rainfall) that has shaped incidence patterns within the area over the past 20 years. *IOI *was used because climate variability in the Indian Ocean is related to global climate change [19] and rainfall has also been evoked to explain cholera epidemic patterns [14,15,20]. We used wavelet analyses and derived methods because these are useful mathematical tools to provide information on the evolution of the periodic component over time and allow quantification of non-stationary associations between time series [21-24].

The new findings provided by this study are useful for the development of an effective early warning system that is based on climate data over an extended intertropical littoral zone. In the future, it will be possible to integrate realtime monitoring of oceanic regions, climate variability and epidemiological and demographic population dynamics to predict cholera outbreaks.

## Methods

### Cholera and climatic data

Epidemiological data corresponding to morbidity reports between 1975 and 2002 were extracted from the Weekly Epidemiological Record published by the World Health Organization (WHO), available on the WHO website [25]. Each report, corresponding to (i) the number of morbidity cases and (ii) the beginning and ending dates of the report, was entered into an internal database. The general characteristics of the morbidity cases dataset are summarized in Table 1. Since the dates between two reports are not uniform, we transformed the data by linear interpolation to obtain monthly data. For example, when the period of WHO report was longer than one month, or began one month and finished the following month or later, e.g., March 15 to April 24, we proportionally attributed the number of morbidity cases over the total period (March 15–April 24) based on the number of days in each of the respective months, i.e., 17 days in March and 24 days in April. Globally, the actual number of cholera cases is known to be much higher; the discrepancy is the result of underreporting and other limitations of surveillance systems, such as inconsistency in case definition and lack of a standard vocabulary. Underreporting may also occur due to fears of unjustified travel and trade-related sanctions [26]. It is therefore paramount that effective public health interventions, such as improved prevention, hygiene and management of the environment, be implemented in order to contain cholera outbreaks among vulnerable populations in high-risk areas. The underreporting poses a serious problem for quantitative analyzes. In this study, we chose to use the wavelet method as a qualitative approach in order to describe the periodicity of cholera epidemics, and thus the results are less affected by underreporting issues.

**Table 1:** Summary of the cholera cases dataset.

Country	Total number of cases (1975–2002)	Total number of reports	Number of reports interpolated (%)	Number of report lengths > 2 months (%)
Côte d'Ivoire	13,887	25	10 (40)	5 (20)
Benin	17,787	78	34 (43.6)	7 (9)
Ghana	55,261	135	64 (47.4)	20 (14.8)
Nigeria	106,272	79	33 (41.8)	16 (20.3)
Togo	9,970	42	25 (59.5)	6 (14.3)

Data were extracted from the Weekly Epidemiological Records published by the World Health Organization for the time period 1975–2002.

Rainfall data were extracted from an historical monthly precipitation data set, 1975 to 1996, available on the Climate Research unit website of the University of East Anglia at Norwich (UK) [27]. Five zones were selected as the most representative of human community settlements, with most of the given country population concentrated near the coastline (see Figure 1). For three of the five countries, we used a mean between rainfall time series because these countries were related to two or more rainfall time series. We computed the mean for (i) Ghana between Ghan132 and Ben270, (ii) Benin between Ben270 and Ben201, and (iii) Nigeria between Ben201 and Nig037 rainfall time series.

The Indian Oscillation Index *(IOI) *[28] is based on the variability in sealevel atmospheric pressure (*SLP*) between Mahe in the Seychelles (4°S, 55°E) in the West Indian Ocean, and Darwin (10°S, 130°E) in the East Indian Ocean. It is realised by computing the differences between the monthly standardized anomalies of *SLP *at both sites (Mahe minus Darwin), from 1975 to 2002. *IOI *warm events (increase in the sea surface temperature and strengthening of easterly winds at the equator) are associated with *IOI *values less than -1. In contrast, values greater than +1 indicate cold events [29].

### Wavelet Analysis: Pattern characterization of Cholera Epidemics and Climate Variability

Wavelet analysis [21,22], in contrast to Fourier analysis, is useful for biological time series analyses, mainly because of the non-stationarity (i.e., the oftenobserved changes in the periodic behaviour) of such series. Wavelet analysis allows detection of periodicity, as well as local variation with time, indicating temporal evolution of the periodic components [22]. Over the past five years, wavelet analysis has increasingly been used in ecology [30,31] and epidemiology [8,21,32,33] to explore spatial and temporal dynamics of disease.

In this study, we used wavelet analysis to determine the significant oscillating modes of disease time series based on wavelet decomposition and wavelet power spectra [22], and we used the phase angles of the disease time series analyses to characterize the pattern of epidemic synchrony [8,21,24,33]. The pattern of disease outbreak synchrony was tested and based on the comparison between the observed distribution of the phase difference and that obtained by bootstrapping. Wavelet coherency analyses identified and quantified possible statistical associations between the two time series, e.g., between the disease time series and climatic indices. Coherency is roughly similar to a classical correlation, but it is relevant to oscillating components in a given frequency mode for a given time period [8,33]. Statistical analyses were performed using Matlab (version 6.5, The MathWorks, Naticks, Massachusetts, United States).

## Results

### Frequency, Synchronicity of Cholera Epidemics, and Climate Variability

**Figure 2:**
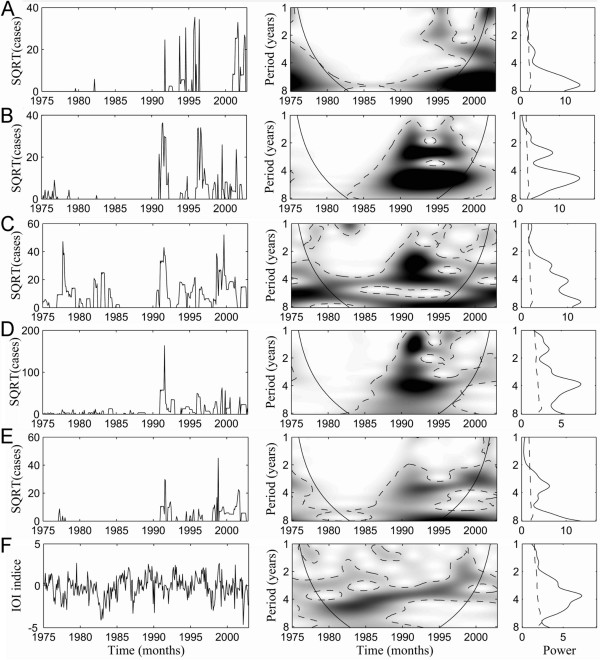
**Wavelet analyses of epidemiological time series for the five countries and Indian Oscillation Index (IOI): (A) Côte d'Ivoire, (B) Benin, (C) Ghana, (D) Nigeria, (E) Togo and (F) IOI**. Prior to wavelet analyses, incidence data were square-root-transformed in order to dampen extremes in variability. In addition, all the time series were normalized. For each time series: (i) the left panel illustrates incidence time series (square root transformed) and climatic index time series; (ii) the middle panel shows the wavelet power spectrum for the different series (xaxis: time in year; yaxis: period in year). The power is coded from low values, in white, to high values, in black. The black dashed lines show the α = 5% significance level computed on 1,000 bootstrapped series. The inside area within the cone of influence (black line) indicates the region not influenced by edge effects. (iii) The right panel corresponds to the global wavelet spectrum (black line) with its significant threshold value of 5% (dashed line).

**Figure 3:**
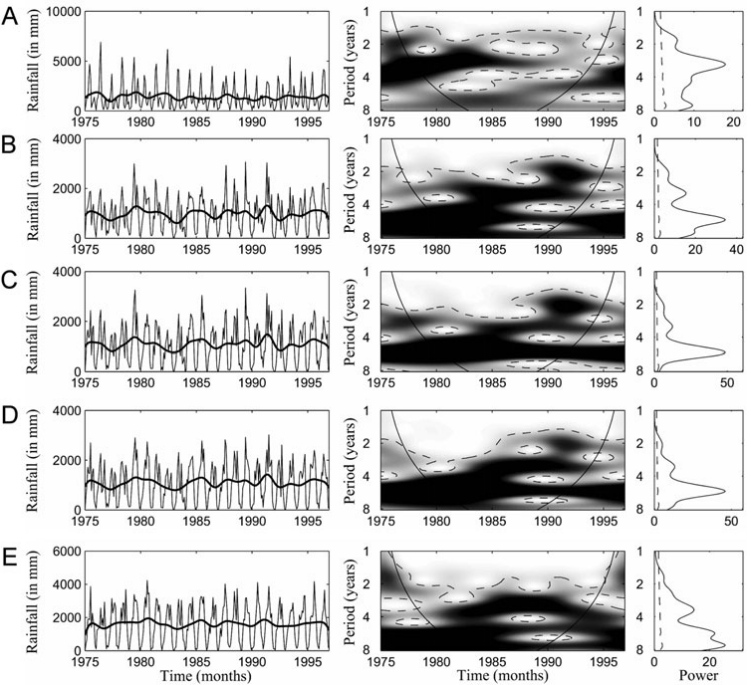
**Wavelet analyses of the rainfall time series: (A) Cotiv021, (B) mean between Ghan132 and Ben270, (C) Ben270, (D) mean between Ben270 and Ben201, and (E) mean between Ben201 and Nige037**. To deseasonalize the rainfall time series we removed all oscillating components with period less than 12 months using a low-pass Gaussian filter. In addition, all time series were normalized. For each time series, (i) the left panel displays rainfall time series. The solid and dense lines correspond to the raw time series and the deseasonalized time series, respectively. (ii) The middle panel shows the wavelet power spectrum for the different series (xaxis: time in year; yaxis: period in year). The power is coded from low values, in white, to high values, in black. The black dashed lines show the α = 5% significance level computed on 1,000 bootstrapped series. The inside area within the cone of influence (black line) indicates the region not influenced by edge effects. (iii) The right panel corresponds to the global wavelet spectrum (black line) with its significant threshold value of 5% (dashed line).

The wavelet and global power spectra analyses of (i) cholera incidence across the five West African countries and *IOI *time series, and (ii) rainfall time series are presented in Figure 2 and in Figure 3, respectively. Periodicities for all incidences and *IOI *time series during 1975–2002 were detected even if transient. Notably, a common 2–5-year periodicity was detected in all countries except for Côte d'Ivoire, and a shift from 4- to 3 year periodicity for *IOI *was found to have occurred between 1989 and 1994. Analyses of rainfall interannual variability between 1975 and 1996 also highlight a common 3–5-year cycle among all countries (Figure 3).

**Figure 4:**
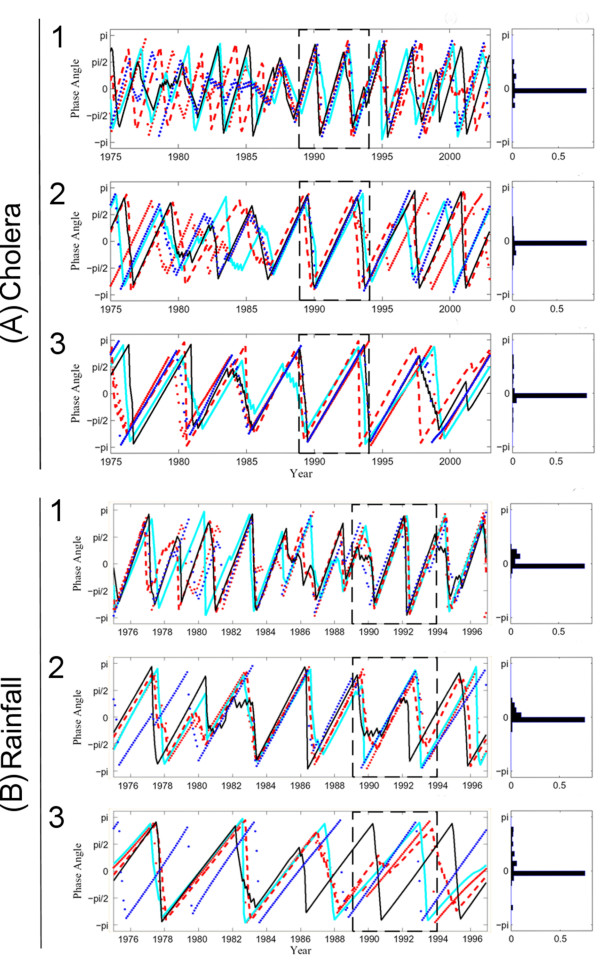
**Pattern of synchrony obtained from phase evolutions of (A) cholera time series between the five countries during 1975–2002 and (B) five selected rainfall time series during 1975–1996**. For the left panels (A): the red dotted line is Benin, the cyan solid line is Côte d'Ivoire, the red dashed line is Ghana, the black solid line represents Nigeria, and the blue dotted line is Togo; (B): the red dotted line is Ben201, the cyan solid line is Ben270, the red dashed line is Ghan110, the black solid line represents Ghan132, the blue dotted line is Cotiv21, and the blue dotted line is Nige037. For both cholera (A) and rainfall (B), the right panels represent the distributions of phase differences between 1989 and 1994, illustrated by a black dashed square in the left panels; analyses were computed with the wavelet transform in three periodic bands: (1) 1.8–2.5 years, (2) 2–3 years, and (3) 4–5 years.

We extracted phase angles in different periodic bands (between 2 and 5 years) to explore the common mode of oscillations of cholera incidence and rainfall. Results (Figure 4-A) show that all incidence time series were synchronous between 1989 and 1994, whatever the explored periodic band, 1.8–2.5-year, 3–4-year, or 4–5-years. Moreover, the observed synchrony between 1989 and 1994 was statistically significant for the three periodic bands (Table 2). Similarly, Figure 4-B shows that all rainfall time series were synchronous during 1989 and 1994 for the 1.8–2.5 and 3–4 year periodic bands (Table 2).

**Table 2:** Results of the test of synchrony for incidence time series and rainfall between 1989 and 1994.

Periodic Band	1.8–2.5 years	3–4 years	4–5 years
Incidence	Entropy = 0.7043 p-value < 0.0001	Entropy = 0.7407 p-value = 0.0004	Entropy = 0.7542 p-value = 0.0360
Rainfall	Entropy = 0.7276 p-value < 0.0001	Entropy = 0.7091 p-value = 0.0041	Entropy = 0.6327 p-value = 0.0669

The test was realized in three different periodic bands and based on normalized entropy, 0 for flat distribution and a 1 for unimodal distribution.

### Association Between Cholera Incidence and Climate Variability

**Figure 5:**
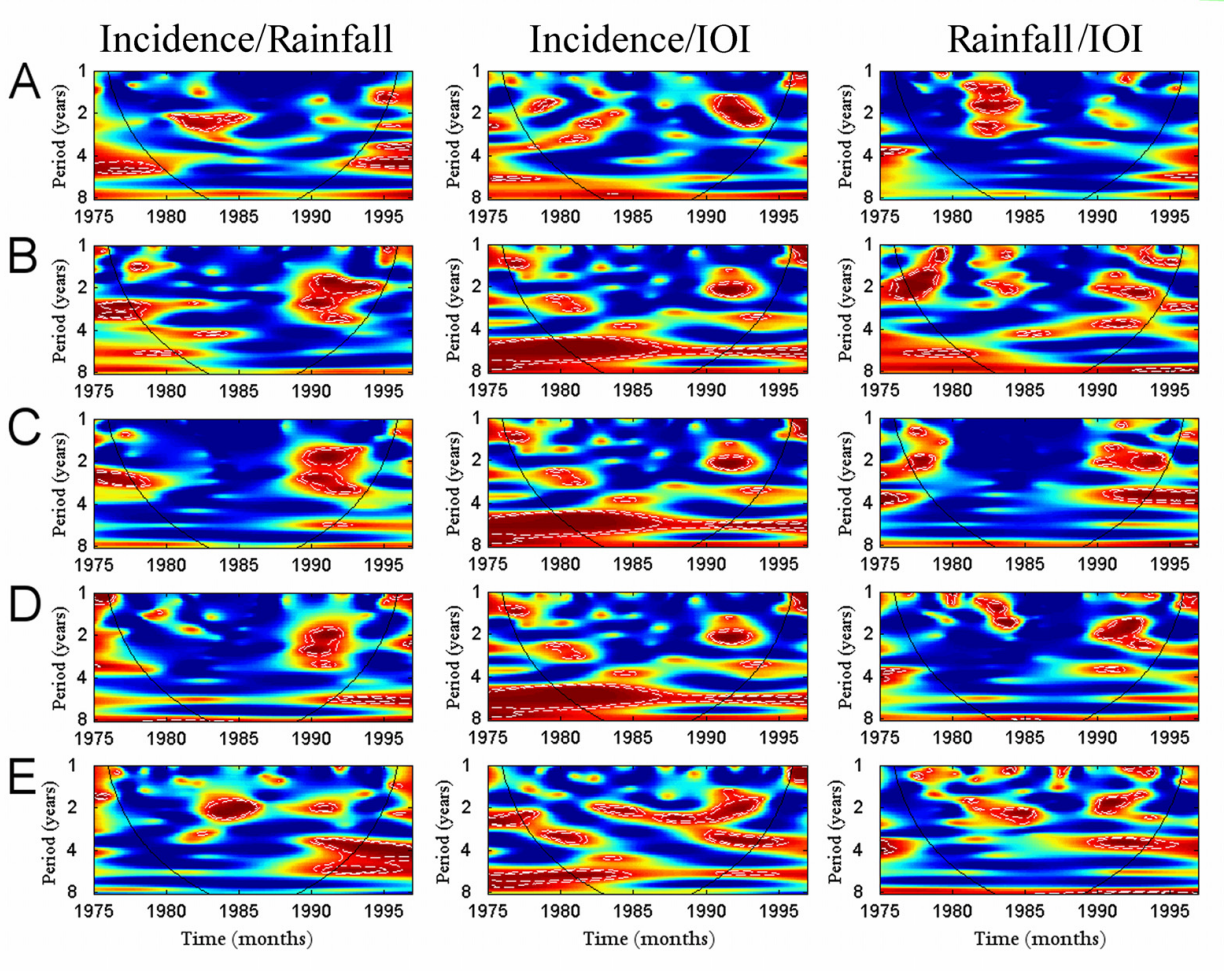
**Coherency analysis between cholera incidence, rainfall, and IOI across five countries: (A) Côte d'Ivoire, (B) Ghana, (C) Togo, (D) Benin, and (E) Nigeria**. For each country, incidence data were square-root transformed (SQRT) to rescale the variance. We obtained mean between selected rainfall time series for three countries: Ghana, mean between Ghan132 and Ben270, Benin mean between Ben270 and Ben201, and Nigeria mean between Ben201 and Nige037. To deseasonalize the rainfall time series all oscillating components with period less than 12 months were removed using a low-pass Gaussian filter. In addition, all series were normalized. Coherency analyses presented are, from left to right: incidence SQRT data *vs *rainfall, incidence SQRT data *vs *IOI, and rainfall *vs *IOI. In the coherence power spectra (x-axis: time in year; y-axis: period in year), power is coded from low value, in dark blue, to high value, in dark red. The white dashed lines show α = 5% and α = 10% significance levels, computed on 1,000 bootstrapped series. The inner area, within the cone of influence (black line), indicates the region not influenced by edge effects.

Wavelet coherency between the time series for the three sets of comparisons, incidence *vs*. rainfall, incidence *vs. IOI*, and rainfall *vs. IOI *are shown for each country in Figure 5. *IOI *and rainfall during the early 1990's were significantly associated with cholera, except for Côte d'Ivoire, in the 2- to 4-year periodic band.

## Discussion

Cholera interannual periodicity and the link between cholera dynamics and climate variability remain incompletely understood and generally focused only on endemic regions [7,9,14,15]. Pascual *et al*. [5] and Rodo *et al*. [7] described a role of El Niño/Southern Oscillation in the dynamics of cholera in Bangladesh. In addition, the complex relationship between largescale climatic variability and spatiotemporal patterns under local environmental conditions and weather contributes to the dynamics of local pathogen populations in aquatic ecosystems [34], and/or disease transmission [35,36]. In this context, using a comparative approach developed for macroecology applications [37], the relationship between cholera incidence in five different African countries and climate interannual variability was explored. Indeed, analyses of long-term monthly disease time series underline both the complex, nonstationary dynamics of cholera epidemics in West Africa, and a relationship with large-scale climate variability.

From 1989 to 1994, (*i*) four of five cholera dynamics (i.e., Benin, Ghana, Nigeria, and Togo), rainfall, and *IOI *displayed a significant 2- to 3-yr periodicity, (*ii*) cholera incidence time series, as well as rainfall time series, were highly synchronous across the five African countries, and (*iii*) the same four of five incidence time series, rainfall and *IOI *were significantly coherent in the 2- to 3-yr periodic band. The 2- to 3-yr periodicity detected in this study was in harmony with results obtained in Asia and in South America [5,7,14]. This remarkable observed synchrony between incidences in the Gulf of Guinea perfectly matches the spatio-temporal synchrony of rainfall, supporting a link between cholera epidemics and climatic variability. Indeed, the influence of rainfall increase on cholera incidence can best be explained by flood waters on disease transmission [15]. Furthermore, the coherency between disease incidence, rainfall and climate variability in the Indian Ocean describes a direct or indirect link, as reported by Rodo *et al*. for Bangladesh.

On the other hand, the Côte d'Ivoire showed no periodicity between 1987 and 1994, and a lack of coherency between incidence and two climatic variables (rainfall and *IOI*). This could be explained by interaction between the two main drivers of the disease, namely, extrinsic factors such as variability in climate or health policy, and intrinsic factors, such as the patterns of immunity in human population. Koelle *et al*. [15] explored interannual cholera cycles in Bangladesh and highlighted the critical interplay of environmental forcing and temporary immunity. Even if local environmental conditions such as rainfall or ambient temperature, influenced by global climate variability, initiate an outbreak, an observed coherency between *IOI *and rainfall in Côte d'Ivoire, the refractory period of the disease dynamic, when the population of susceptibles is low, can prevent outbreaks. Furthermore, sanitary conditions and access to health care centers reduce the susceptible population size and transmission probability, thus resulting in a decline in the sensitivity of cholera dynamics to climate variability.

In fact, the question raised from the results of this study concerns the dynamics of cholera: Is synchronization of cholera a response of local populations, according to ecological theory, to climate interannual variability? The presence of global synchrony among the countries for cholera, a disease highly susceptible to climatic factors, supports the hypothesis of a common external forcing, namely climatic factors, explaining synchronicity, and supporting a key prediction of the Moran theorem [38], a phenomenon described in population biology [38-41] and in epidemiology [8,42]. The hypothesis consists of an intricate, hierarchical mechanism with climatic variability at a large scale quantified by *IOI *at the origin of the synchronization of both the cholera incidence and rainfall over all of the West African countries included in this study. Indeed, Janicot (1997) described that empirical studies have shown that warm El Niño/Southern Oscillation (ENSO) episodes are associated with the intertropical convergence zone (ITCZ) over the tropical Atlantic which is related to the rainfall in West Africa. It is also likely that many other factors, e.g., the level of poverty and human population density, influence the spatial and temporal distribution of cholera [13,36,43], but perhaps now, cholera dynamics are more strongly associated with climate [9,14,44,45]. [46]

## Conclusion

In conclusion, besides the two inter-tropical regions of the world, Asia and South America, global climate change may well impact cholera diseases in many other parts of the inter-tropical zone [5,7,8,14,15]. The study reported here is an important step toward a long-term study of the spatial and temporal dynamics of cholera in Africa at the regional level. The main perspective of this work should be to focus on a local scale, as has been done in Bangladesh, using collected data from diarrhoeal surveillance programs in selected African areas. The benefit of the precision of this type of study will open a new field of research and allow a reliable model for cholera predictions [14,15,45,47,48]. Development in the near future of a concrete and useful plan of action for health policy should be based coupling both realistic epidemiological models, including intrinsic factors such as level of immunity or cross immunity of the population, and environmental parameters monitored by remote sensing, such as sea surface temperature, sea surface height or land surface temperature [10,15,49].

## Competing interests

The author(s) declare that they have no competing interests.

## Authors' contributions

GCdM contributed to the conception and design of the study, to acquisition of data, performed the statistical analysis and interpretations, and wrote the draft and final versions of the manuscript. J-FG participated in the conception and design of the study and in the acquisition of funding, and helped write the draft and final versions of the manuscript. MP helped to acquire data and to draft the manuscript. BC contributed to the conception and design of the study, helped to perform the statistical analyses and interpretations, and helped write the draft and final versions of the manuscript. All authors read and approved the final manuscript.

## Pre-publication history

The pre-publication history for this paper can be accessed here:


